# Bibliography

**Published:** 1902-07

**Authors:** 


### Bibliography.

ABBOTT'S BACTERIOLOGY. A Practical Manual of Bacteriology for Students and Physicians. By A. C. Abbott, M.D., Professor of Hygiene, University of Pennsylvania. New Sixth Edition, revised and enlarged. In one 12mo. volume of six hundred and thirty-six pages, with one hundred and eleven illustrations, of which twenty-six are colored. Lea Brothers & Co., Publishers, Philadelphia and New York.

The issue of the sixth edition of this most valuable work on Bacteriology, by Dr. Abbott, is the best evidence, if any such be needed, that the author has met a want and has given the seekers after information in this direction a book at once reliable and as thorough as present experimental knowledge can produce.

The fifth edition of this book was fully reviewed in this journal some three years since, and the favorable opinion then expressed can be repeated, with this addition, that the sixth has been greatly improved and made somewhat larger by the advances made through bacteriological researches.

The author says, in the "Preface to the Sixth Edition," "The chapter on suppurative and inflammatory conditions has been enlarged with a description of the causative agent of epidemic cerebrospinal meningitis; that on tuberculosis with a summary of our knowledge of the acid-resisting bacilli that are in many ways allied to bacillus tuberculosis, and with a description of the more important pathogenic streptothrices; to the chapter relating to the infections of the intestinal canal a summary of the new work on dysentery, with a description of the micro-organism now believed to be the cause of that disease, has been added."

Dr. Abbott has answered by the book itself the query which he says the student invariably asks after one observation has been completed: "What shall I do next?" The book satisfies this natural inquiry very fully, for after defining what is meant by bacteria and "their place in nature," morphology, etc., he carries the reader step by step through the technic necessary in the study of these low forms of life, and from the practical he advances to the "Application of the Methods of Bacteriology. Descriptions of the more Important Species."

Chapter XVIII. deals with "Tuberculosis, Microscopic Appearance of Miliary Tubercles, etc.," and is one of the most important in the book. It is, however, impossible to give a special place to any one chapter as being more important than another. The reader will find each pathological subject treated with that clearness of description that has made this book of Dr. Abbott valuable above others of a similar character. The feeling goes with the reading that all that is known to date regarding bacteria has been mastered by the author, and by careful study of its contents the student may share in degree an equal knowledge.

While the bacteriological laboratory cannot take the place of clinical experience, the practitioner who depends solely on symptomatology for his information and not upon the origin of pathological conditions cannot be one to be relied upon. On the other hand, laboratory work alone is not sufficient to make the skilled diagnostician. Hence the true pathologist combines laboratory and clinical experience.

For those who have not had the opportunity for laboratory research, and they are legion, this book points the way to become intelligent upon a subject one of the most important of all modern medical studies, and this is especially true as applied to dentistry. The oral cavity is the vestibule of the entire organism, and the pathogenic and non-pathogenic organisms contained therein demand special study, and one unfamiliar with these has no right to assume the responsibilities with which he is hourly and daily confronted. To such as these a careful study of this book will be of lasting benefit, and to all others it will be a constant and invaluable work of reference.

NOTES ON MATERIA MEDICA, PHARMACOLOGY, AND THERAPEUTICS, FOR DENTAL STUDENTS AND PRACTITIONERS. By Douglas Gabell, L.R.C.P., M.R.C.S., L.D.S., Assistant Dental Surgeon Royal Dental Hospital, and Harold Austen, M.D., B.S. (Lond.), L.R.C.P., M.R.C.S., L.D.S., Assistant Dental Surgeon Royal Dental Hospital and Lecturer on Dental Materia Medica. Claudius Ash & Sons, Limited, London, 1902.

There is a great need of a work on materia medica designed exclusively for dental students. Attempts have been made to meet this demand, with more or less success, but the ideal text-book on this subject has not yet appeared.

When this book was opened for review it was with the hope that at last this had been accomplished. The disappointment was therefore great, on reading the preface, to find the authors writing as follows: "It will hardly be said that this volume does not claim to be a complete treatise upon the so-called dental materia medica, nor, indeed, anything more than an attempt to set down as briefly and in as practical a manner as possible those properties, actions, and applications of drugs which are of utility or interest to the dental surgeon.

"For this reason any description of the *general* therapeutic action of drugs has been omitted, except in cases . . . such therapeutic action approaches nearly to the border-line which . . . divides dental from general medical practice."

It is difficult to imagine that a work on materia medica can have any real practical value that omits allusion to the therapeutic value of the drugs described. It is the fault of all works of this character that the authors seem to consider condensation a great virtue. This is no doubt a valuable quality, but when writers on materia medica try to be as brief as possible, it means a mere skeleton of facts, the form of which is to be memorized by the student. There is no room here for that mental analyzation so necessary in this study.

The authors consider the "pharmacological classification of drugs" to be the most practical one for the dentist. This has an advantage over that ordinarily used, but it means much cross reference that must become annoying to the student, and it also involves constant repetition, which, while not objectionable as a means of impressing facts, becomes perplexing when searching for them.

It would be well in future editions to omit this sentence: "The caustic action of arsenic may be employed as an obtundent of sensitive dentine." The authors say subsequently, "It [arsenic] is very effective, but very dangerous to the pulp." If so, and there is not the slightest doubt of the truth of this remark, why use it at all? Experience has demonstrated that the pulp will become devitalized by its use, no matter how shallow the cavity.

In many respects this book is an improvement over others written upon this subject, and should the authors change the objectionable features and add some matter omitted, it may be made one of the most valuable text-books published, for it is not only well written, but generally very correct, a very important matter in a book of this kind and a quality unfortunately not always present.

